# Tomatidine Suppresses the Destructive Behaviors of Fibroblast-Like Synoviocytes and Ameliorates Type II Collagen-Induced Arthritis in Rats

**DOI:** 10.3389/fphar.2021.670707

**Published:** 2021-08-26

**Authors:** Xiaolu Yu, Junnan Zhou, Fuli Zhao, Xuan Liu, Yuhang Mao, Li Diao, Chuanjun Wen, Mei Liu

**Affiliations:** Jiangsu Key Laboratory for Molecular and Medical Biotechnology, College of Life Sciences, Nanjing Normal University, Nanjing, China

**Keywords:** tomatidine, rheumatoid arthritis, fibroblast-like synoviocytes, collagen-induced arthritis, MAPK, NF-κB

## Abstract

Fibroblast-like synoviocytes (FLSs) are the prominent non-immune cells in synovium and play a pivotal role in rheumatoid arthritis (RA) pathogenesis. Searching for natural compounds that may suppress the pathological phenotypes of FLSs is important for the development of RA treatment. Tomatidine (Td), a steroidal alkaloid derived from the solanaceae family, has been reported to have anti-inflammatory, anti-tumor and immunomodulatory effects. However, its effect on RA remains unknown. Here, we examined the inhibitory effect of Td on TNFα-induced arthritic FLSs, and subsequently investigated its therapeutic effect on collagen-induced arthritis (CIA) rats. Our results revealed that Td significantly inhibited TNFα-induced proliferation and migration of arthritic FLSs. In addition, we found that Td treatment could efficaciously ameliorate synovial inflammation and joint destruction of rats with CIA. Both *in vitro* and *in vivo* studies showed that Td significantly suppressed the production of pro-inflammatory cytokines including IL-1β, IL-6 and TNFα, and downregulated the expression of MMP-9 and RANKL. Further molecular mechanism studies revealed that the inhibitory effect of Td on RA might attribute to the decreased activations of MAPKs (ERK and JNK) and NF-κB. These findings provide evidence that Td has the potential to be developed into a complementary or alternative agent for RA therapy.

## Introduction

Rheumatoid arthritis (RA) is a chronic autoimmune inflammatory disorder characterized byinflammation of the synovium and destruction of bone tissue (Ath and Denton, 1991; Athanasou, 1996; Danks et al., 2002). Although RA pathogenesis remains incompletely understood, many cell types such as fibroblast-like synoviocytes (FLSs), macrophages, osteoclasts and lymphocytes have been demonstrated to be involved (Gravallese, 2002; Ma and Pope, 2005; Skapenko et al., 2005). As the important effector cells in this disease, FLSs exhibit tumor-like characteristics such as overproliferation, aggressive migration and invasion, and apoptosis-resistance (Neidhart et al., 2003; Mor et al., 2005; Noss and Brenner, 2008; Bartok and Firestein, 2010; Lahoti et al., 2014). These over-activated FLSs can produce multiple pro-inflammatory cytokines which promote the formation of pannus and the destruction of cartilage and bone (Firestein, 2003). In addition, the pathogenic properties of FLSs have been demonstrated to tightly correlate with histological and radiographic damage in RA animal models and patients (Lange et al., 2005; Laragione et al., 2008). Therefore, FLS-targeted therapy represents an attractive complementary approach to immune-directed therapies in RA.

Many current drugs used for RA therapy have relatively limited effectiveness and unwanted side effects. Therefore, botanical medicines as alternative remedies have become increasingly popular as they are believed to be safe, efficacious and have over a thousand years’ history in treating patients. Furthermore, analysis of patents on anti-RA therapies issued in China revealed that traditional Chinese Medicine may provide substantial new information for anti-RA drug development (Yuan et al., 2015). Tomatidine (Td) is a natural steroidal alkaloid that isolated from the Solanaceae plants such as tomatoes, potatoes and eggplants (Lange et al., 2005; Laragione et al., 2008). Steroidal alkaloids are known to be essentially nitrogen analogues of steroid saponins such as diosgenin, which is a precursor of steroidal hormones and anti-inflammatory steroids. Nowadays, Td has received increasing attention for its confirmed pharmacological safety and a variety of biologic activities, including cardioprotective, antioxidative, anti-inflammatory, anticancer, anti-osteoporosis, and immunoregulatory effects, as well as its ability to inhibit muscle atrophy (Chiu and Lin, 2008; Friedman et al., 2009; Lee et al., 2011; Shieh et al., 2011; Yan et al., 2013; Hu et al., 2019; Jeon and Kim, 2019). For example, Chiu and Lin found that Td effectively inhibited the expressions of COX2 and iNOS by suppressing NF-κB and JNK pathways in LPS-stimulated mouse macrophage cells (Chiu and Lin, 2008). Meanwhile, Td was demonstrated to regulate the function of the immune system through stimulating potent antigen-specific humoral and cellular immune responses (Lee et al., 2004). In addition, Td could effectively inhibit the proliferation and invasion of tumor cells, and promote the cell apoptosis (Friedman et al., 2009; Lee et al., 2011; Shieh et al., 2011). More importantly, Hu et al. found that Td suppressed osteoclast formation and mitigated estrogen deficiency-induced bone mass loss (Hu et al., 2019). Given the reported pharmacological functions of Td and the clinical and pathological features of RA, we hypothesized that Td might represent a novel treatment for RA. Thus, we here investigated the therapeutic effect of Td on cultured arthritic FLSs and type Ⅱ collagen-induced arthritis (CIA) in rats, and subsequently explored their underlying mechanisms.

## Materials and Methods

### Reagents

Td (C₂₇H₄₅NO₂, purity ≥ 98%) was obtained from MedchemExpress (Shanghai, China). Lyophilized native chicken type Ⅱ collagen (CⅡ) was purchased from Chengdu Herbpurify Co., Ltd. (Chengdu, Sichuan, China). Methotrexate (MTX) was from Shanghai Sine Pharmaceutical Co., Ltd. (Shanghai, China). Commercial kits for measurement of alanine aminotransferase (ALT) and aspartate aminotransferase (AST) were from Jiancheng Institute of Biotechnology (Nanjing, Jiangsu, China). IL-1β, IL-6 and TNFα ELISA kits were purchased from SenBeiJia Biological Technology Co., Ltd. (Nanjing, Jiangsu, China). Dulbecco’s modified Eagle’s medium (DMEM) was purchased from Gibco (Gibco BRL, Grand Island, NY, United States). Fetal bovine serum (FBS) was purchased from Invigentech (Sydney, Australia). Recombinant tumor necrosis factor alpha (TNFα) was purchased from Peprotech (PeproTech, Rocky Hill, NJ, United States). MTS reagents, Triton-X 100, DAPI solution and Freund’s complete adjuvant (CFA) were purchased from Sigma-Aldrich (St Louis, MO, United States). 5-Ethynyl-2ʹ-deoxyuridine (EdU) was obtained from Guangzhou RiBo Bio Co., Ltd. (Guangzhou, Guangdong, China). Annexin V-FITC apoptosis detection kit was from KeyGEN Biotech Co., Ltd. (Nanjing, Jiangsu, China). TRIzol reagent was from Invitrogen Life Technologies (Carlsbad, CA, United States). Primary antibodies targeting phosphorylated extracellular signal-regulated kinase 1/2 (p-ERK1/2), phosphorylated c-Jun N-terminal kinases (p-JNK), phosphorylated p38 (p-p38), phosphorylated inhibitor of NF-κB (p-IκBα), total ERK, total JNK, total p38, IκBα, p65, MMP-2, MMP-9 and glyceraldehyde-3-phosphate dehydrogenase (GAPDH) were purchased from CST (Cell Signaling Technology, Inc., Beverly, MA, United States). Anti-RANKL antibody was obtained from Proteintech (Proteintech Group, Inc., Chicago, United States).

### Isolation and Culture of Arthritic Fibroblast-Like Synoviocytes

Arthritic FLS cells were isolated and cultured as previously reported (Liu Z. et al., 2018). Briefly, the synovial tissues were dissected from the knees of the vehicle-treated CIA rats, minced and incubated with 0.25% trypsase and 0.4% collagenase. Arthritic FLS cells were collected and cultured in DMEM supplemented with 10% FBS, 100 U·mL^−1^ penicillin and 100 μg·mL^−1^ streptomycin at 37°C in 5% CO_2_. Cells from passages three to nine were used for all *in vitro* experiments.

### Cell Viability Assay

Arthritic FLS cells were seeded into 96-well plates and cultured for 48 h with complete DMEM containing varying concentrations of Td (0, 2.5, 5, 10, 20, 40, 80 μM). MTS/PMS mixture was added and cells were incubated for another 4 h. The absorbance at 490 nm was measured using a microplate reader (Model 680, BioRAD, Hercules, CA, United States). The effect of Td on cell viability was expressed as percent cell viability with vehicle treated control cells set at 100%. GraphPad Prism (version 5.0c; San Diego, CA) was used to calculate the half maximal inhibitory concentration (IC_50_).

### Ethynyl-2ʹ-deoxyuridine Incorporation Assay

Arthritic FLS cells were incubated with Td (5 μM) for 6 h and then stimulated with or without TNFα (50 ng·mL^−1^) for 24 h. EdU (10 μM) was added to measure the newly synthesized DNA and Hoechst 33342 was used to counterstain cell nuclei. The cell proliferation rate was calculated as the proportion of nucleated cells incorporating EdU in five high-power fields per well.

### Wound-Healing Assay

Wound-healing assay was carried out as our previous descriptions (Liu et al., 2018b; Qiu et al., 2018). In brief, arthritic FLSs were seeded into 12-well plates and cultured to 80% confluency. A linear scratch was created using a sterile 200 ul pipette tip. After washing the suspended cell debris with PBS, arthritic FLSs were treated with different concentrations of Td (0, 2.5, 5 and 10 μM) for 1 h and then stimulated with TNFα (50 ng·mL^−1^) for 24 h. The cell migration area was measured by comparing the remaining cell-free area in the identical fields using Image J software (National Institutes of Health, Bethesda, MD, United States).

### Apoptosis Assay

Arthritic FLS cells were seeded into 6-well plates and incubated with varying concentrations of Td (0, 2.5, 5 and 10 μM) for 24 h. Cells were then harvested for staining with annexin V and propidium iodide (PI) solution. The cells were detected by a flow cytometer (Beckman Coulter, CA, United States) and the apoptotic cell percentage was analyzed using Cell Quest Software (FACScan; Becton Dickinson, Franklin Lakes, NJ, United States).

### Cytokine and MMP Analysis in Cultured Arthritic Fibroblast-Like Synoviocytes

Arthritic FLS cells were incubated with varying concentrations of Td (0, 2.5, 5 and 10 μM) for 1 h and then stimulated with TNFα (50 ng·mL^−1^) for 24 h. The cells were harvested for real-time polymerase chain reaction (PCR) assay or Western blot analysis. The culture supernatants were collected for ELISA assay.

For real-time PCR assay, the total RNA was extracted using TRIzol reagent and subjected to cDNA synthesis according to the manufacturer’s instructions. Real-time PCR was performed using a SYBR Green PCR Kit (Vazyme Biotech) and run in Mastercycler ep realplex two systems (Eppendorf, Hamburg, Germany). The primer pairs for *IL-1β*, *IL-6*, *TNFα*, *MMP-2*, *MMP-9*, *RANKL* and *β-actin* were used as the previous descriptions (Ueland et al., 2005; Liu et al., 2018b). The expression of each target gene was normalized to the transcription level of *β-actin* gene.

For Western blot assay, the cells were lysed and the protein levels of MMP-2, MMP-9 and RANKL were examined by Western blot as described below.

For ELISA assay, the culture supernatants were centrifuged at 2000 rpm for 20 min to remove the particulate matter. The pro-inflammatory cytokines IL-1β, IL-6 and TNFα were determined using cytokine-specific ELISA kits according to the manufacturer’s instructions.

### Collagen-Induced Arthritis

A total of 30 female Wistar rats (170–180 g, about 6–8 weeks) were purchased from Beijing Vital River Laboratory Animal Technology Co., Ltd. (Beijing, China). All of the rats were housed under specific pathogen-free (SPF) conditions (22°C, 12 h/12 h light/dark, 50–55% humidity) and given free access to food and water. All the animal experiments were approved by the Experimental Committee of Nanjing Normal University (#2020065, approved date July 15, 2020).

The CIA model was induced as previously described (Liu et al., 2018a; Liu et al., 2018b; Qiu et al., 2018). Briefly, the rats were intradermally injected with 1 mg native chicken CII emulsified in Freund’s complete adjuvant. Seven days later, the rats were subcutaneously boosted with half the amount of CII emulsified in Freund’s incomplete adjuvant. The normal control rats were immunized with saline (*n* = 6). All the immunized rats developed CIA (clinical score ≥2) after a mean (±SD) interval of 14 ± 1 days. Rats with CIA were randomly divided into four groups as two Td-treated groups, MTX-treated group and vehicle-treated group (*n* = 6 per group). The MTX-treated group was administrated with MTX (3 mg·kg^−1^ body weight) every 3 days (according to clinical usage). The Td-treated groups were intraperitoneally injected with different doses of Td (5 and 15 mg·kg^−1^ body weight, respectively) and the vehicle-treated group was injected with 0.9% saline every day for a 14-day period. The doses of Td were determined according to the previous reports (Fujiwara et al., 2012; Dyle et al., 2014; Hu et al., 2019) with modification from our preliminary experiments. Clinical arthritic scores were evaluated using a scoring system of 0–4 for each limb: 0 = no swelling; 1 = swelling and/or redness of one to two interphalangeal joints; 2 = involvement of three to four interphalangeal joints or one larger joint; 3 = more than four joints red/swollen; 4 = severe arthritis of an entire paw. The reported arthritis score for each rat was the sum of the two hind paws. The volumes of the hind paws were measured using a volume displacement plethysmometer (YLS-7A, Facility Station of Shandong Academy of Medical Science, Shandong, China). Arthritis scoring and paw volume measurements were performed by two independent observers in a blinded manner. After a 14-days treatment, serum was collected for measurement of pro-inflammatory cytokines, ALT and AST. Joint tissues were harvested for radiographic and histopathological evaluation, cytokine and MMP analysis, and signaling pathway detection. In addition, we weighed the main organs including heart, liver, kidney, spleen and thymus, and calculated the organ coefficients, as our previous report (Liu et al., 2013). Also, the change in body weight of each individual CIA rat was calculated as the following formula: change in body weight = [(body weight of day 14 arthritis/body weight of day 1 arthritis)−1] *100%.

### Radiographic Evaluation

The left hind paws were separated, fixed and exposed under X-ray (MX-20; Faxitron X-ray Corporation, Wheeling, IL, United States). Bone destruction was evaluated using a scoring system of 0–3, as the previous report (Cai et al., 2007). The radiological evaluation was carried out by two independent observers in a blinded manner.

### Histopathological Assessment

After X-ray examination, the left ankle joints were decalcified in 12% ethylenediaminetetraacetic acid (EDTA) and then embedded in paraffin. Sections with a thickness of 4 μm were stained with haematoxylin and eosin (H&E). Pathological changes in inflammation, pannus formation, synovial hyperplasia, cartilage destruction and bone erosion were scored using a semi-quantitative scale, as our previous descriptions (Liu et al., 2018a; Liu et al., 2018b; Qiu et al., 2018). Histopathological analysis was recorded by two independent observers in a blinded manner.

### Cytokine and MMP Analysis in Collagen-Induced Arthritis Rats

Serum was collected by centrifugation (5000 rpm for 15 min) and stored at −80°C until analysis. The levels of IL-1β, IL-6 and TNFα were measured using ELISA kits according to the standard method. In addition, serum ALT and AST were detected using commercial kits following the protocol of manufacturer’s instructions.

The right hind paws of rats were removed and homogenized, as our previous report (Liu et al., 2018a; Liu et al., 2018b; Qiu et al., 2018). The homogenates were used to detect the protein levels of IL-1β, IL-6, TNFα and MMP-9, RANKL by ELISA and Western blot, respectively. In addition, the phosphorylation changes of MAPKs (ERK, JNK and p38) and the protein level of IκBα were examined by Western blot.

### Western Blot Analysis

Arthritic cells or joint tissues were homogenized in radioimmunoprecipitation assay buffer and the supernatants containing proteins were collected by centrifugation. Protein was separated by sodium dodecyl sulphate–polyacrylamide gelelectrophoresis (SDS-PAGE) and then transferred to nitrocellulose membranes. The membranes were incubated with various kinds of primary antibodies including p-JNK, JNK, p-ERK, ERK, p-p38, p38, p-IκBα, IκBα, MMP-2, MMP-9, RANKL and GAPDH. The immunoreactivity was visualized using enhanced chemiluminescence reagents (Labgic Technology) according to the manufacturer’s instructions. Three independent experiments were performed and the intensity of each band was analyzed using ImageJ software.

### Confocal Microscopy for NF-κB Localization

Arthritic FLS cells were seeded on sterile cover slips in a 24-well plate and cultured overnight at 37°C. Following serum starvation, the cells were treated with Td (10 μM) for 4 h, and then stimulated with TNFα (50 ng·mL^−1^) for 30 min. After washing with PBS, the cells were fixed by methanol and permeabilized by 0.5% Triton-X 100. The arthritic FLS cells were blocked with 10% goat serum and then incubated with NF-κB p65 antibody. DAPI solution were used to stain cell nuclear. The nuclear translocation of p65 was imaged using a Nikon A1R resonance scanning confocal microscope with spectral detector (Nikon, Tokyo, Japan).

### Statistical Analysis

All data were expressed as the mean ± SD of results obtained from three or more experiments. Multiple comparisons were performed using one-way analysis of variance (ANOVA), followed by Tukey’s p*ost hoc* analysis. Comparisons between two groups were made using Student’s *t*-test. *P* < 0.05 was considered statistically significant.

## Results

### Tomatidine Suppressed TNFα-Induced Proliferation and Migration of Arthritic FLSs

**FIGURE 1:**
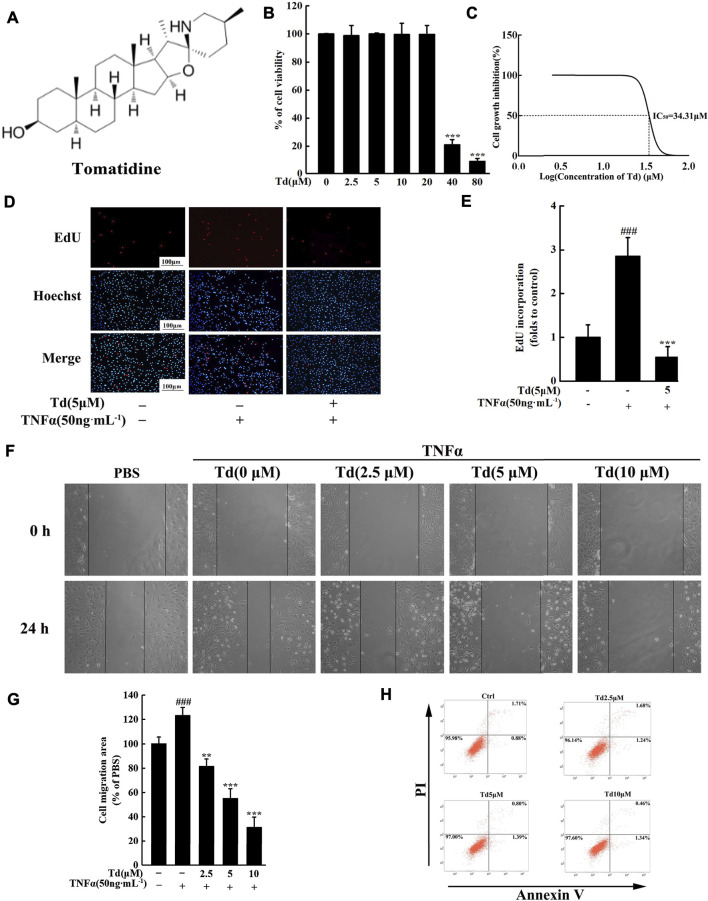
Td attenuated TNFα-induced proliferation and migration of arthritic FLS *in vitro*. **(A)** The chemical structure of Td. **(B,C)** The viability of Td-treated arthritic FLSs was quantified using an MTS assay and the IC_50_ was calculated. **(D,E)** The EdU assay showed that Td (5 μM) significantly inhibited TNFα-induced proliferation of arthritic FLSs. ^**###**^ *P* < 0.001 versus TNFα-untreated, Td-untreated cells; ^***^ *P* < 0.001 versus TNFα-treated, Td-untreated cells. **(F,G)** Td dose-dependently reduced TNFα-induced migration of arthritic FLSs. Serum-starved cells were treated with various doses of Td (0, 2.5, 5 and 10 μM) for 1 h and then incubated with TNFα (50 ng·mL^−1^) for 24 h. The cell migration rate was measured by a wound-healing assay. ^**###**^ *P* < 0.001 versus TNFα-untreated, Td-untreated cells; ^**^ *P* < 0.01 and ^***^ *P* < 0.001 versus TNFα-treated, Td-untreated cells. **(H)** After treating with varying doses of Td (0, 2.5, 5, and 10 µM) for 24 h, the arthritic FLSs were stained with Annexin V-FITC and propidium iodide (PI). Flow cytometry was used to detect the percentage of apoptosis cells within each population.

Arthritic FLSs are the main effector cells of RA. Inhibiting the over-activated properties of FLSs has become a potential approach to RA therapies. In this study, we firstly used an MTS assay to quantify the potential cytotoxicity of Td on FLSs. As shown in Figure 1B, up to 20 μM for 2 days treatment, arthritic FLSs did not show a significant decline in cell viability. And the calculated IC_50_ was 34.31 μM (Figure 1C). In the subsequent *in vitro* experiments, concentrations of Td used did not exceed 10 μM. We here investigated the effects of Td on TNFα-induced proliferation, migration and apoptosis in arthritic FLSs. The EdU assay showed that TNFα stimulation could dramatically enhance FLS’s proliferative potential, however, this enhancement was significantly suppressed by 5 μM Td (Figures 1D,E). The wound healing assay demonstrated that Td at doses of 2.5–10 μM could significantly and dose-dependently inhibit the migration rate of arthritic FLSs (Figures 1F,G). Since Td was able to induce apoptosis in several tumor cells (Song et al., 2018), we analyzed the apoptosis-inducing effect of Td on arthritic FLSs. As shown in Figure 1H, the given doses of Td had little effect on the apoptotic rate of arthritic FLSs, suggesting that the suppression of Td on proliferation and migration was not due to its apoptosis-inducing action.

### Tomatidine Decreased the Production of Cytokines and MMP-9 in TNFα-Stimulated Arthritic FLSs

**FIGURE 2:**
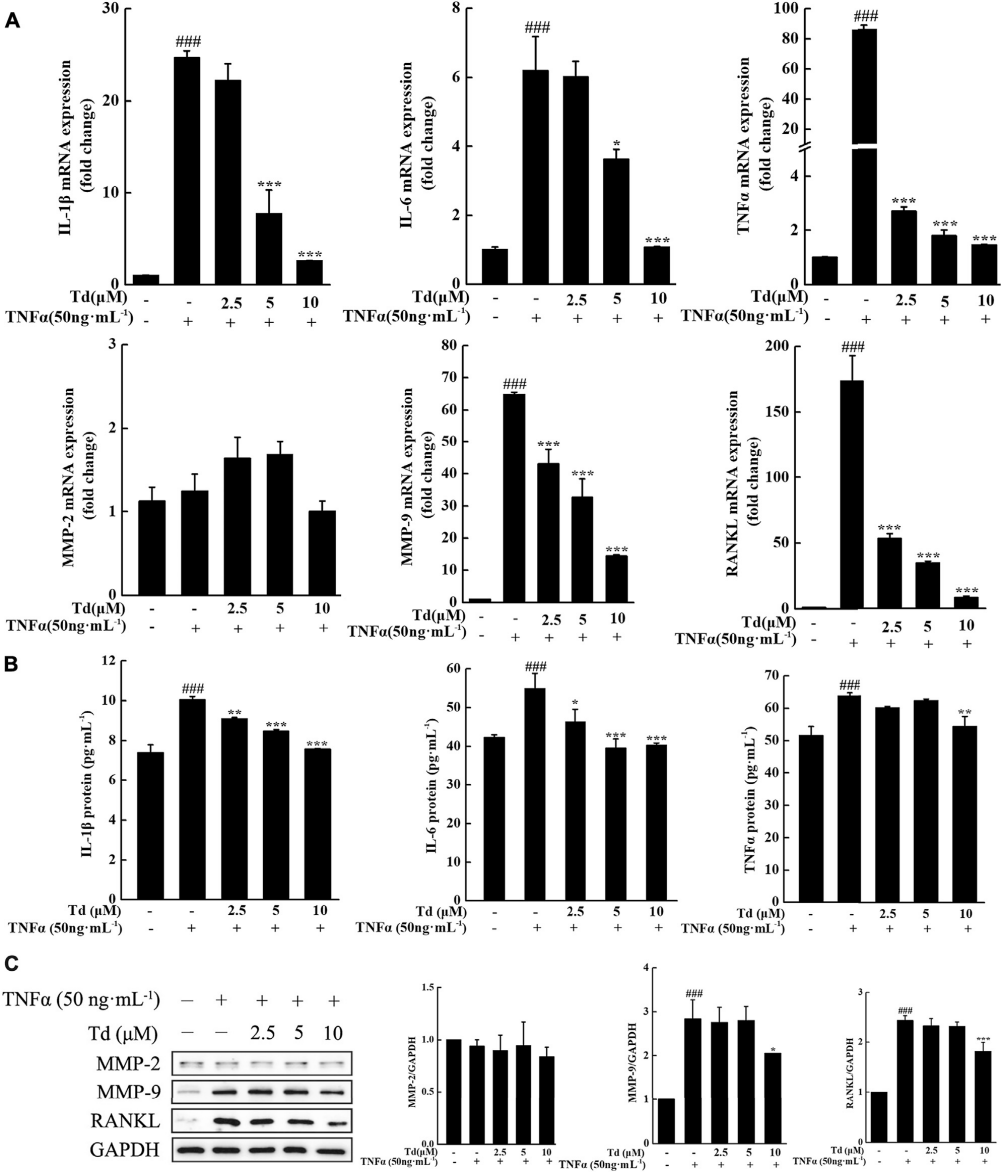
Td decreased the production of proinflammatory cytokines, RANKL and MMP-9 in TNFα-induced arthritic FLSs. Arthritic FLSs were treated with various doses of Td (0, 2.5, 5 and 10 μM) for 1 h and then incubated with TNFα (50 ng·mL^−1^) for 24 h. The cells were harvested for real-time polymerase chain reaction (PCR) assay or Western blot analysis. The culture supernatants were collected for ELISA assay. **(A)** The total RNA was extracted and real-time PCR was performed to measure the transcripts of *IL-1β, IL-6*, *TNFα*, *MMP-2*, *MMP-9* and *RANKL*. The mRNA levels of these genes were normalized to *β-actin* and represented as fold change over the TNFα-untreated, Td-untreated cells. *n* = 3, ^**###**^ *P* < 0.001 versus TNFα-untreated, Td-untreated cells; ^*^ *P* < 0.05 and ^***^ *P* < 0.001 versus TNFα-treated, Td-untreated cells. **(B)** The culture supernatants were collected and ELISA was performed to detect the protein levels of IL-1β, IL-6, TNFα. *n* = 3, ^**###**^ *P* < 0.001 versus TNFα-untreated, Td-untreated cells; ^*^ *P* < 0.05, ^**^ *P* < 0.01 and ^***^ *P* < 0.001 versus TNFα-treated, Td-untreated cells. **(C)** Western blot was performed to examine the protein expressions of MMP-2, MMP-9 and RANKL. Relative expression was determined by densitometric analysis using ImageJ software. *n* = 3, ^**###**^ *P* < 0.001 versus TNFα-untreated, Td-untreated cells; ^*^ *P* < 0.05 and ^***^ *P* < 0.001 versus TNFα-treated, Td-untreated cells.

It is well known that RA is an immune-driven inflammation disease and manifests as cartilage and bone erosion occurs the later stage of the disease. Efforts should be made to inhibit the expressions of pro-inflammatory cytokines as well as genes involved cartilage and bone destruction in RA treatment. Our studies revealed that TNFα stimulation could dramatically upregulate the mRNA levels of pro-inflammatory cytokines including *IL-1β*, *IL-6* and *TNFα*. However, these upregulations were significantly inhibited by Td treatment (Figure 2A). Consistent with the real-time PCR data, ELISA assay showed that the protein levels of IL-1β, IL-6 and TNFα were also decreased by Td (Figure 2B), which further confirmed the anti-inflammatory action of Td. Rheumatoid FLSs contribute to the degradation of connective tissue by secreting substantial amounts of MMPs, which results in the breakdown of cartilage destruction. In this study, the data of real-time PCR and Western blot both demonstrated that Td had no obvious effect on the expression of MMP-2 but distinctively inhibited MMP-9 production (Figures 2A,C). RANKL has been regarded as the strongest inducer in osteoclast differentiation and activity (Yasuda et al., 1998; Kobayashi et al., 2000; Liu et al., 2018a). In our study, RANKL was significantly suppressed by Td at both the mRNA and protein levels (Figures 2A,C), indicating that Td might play an important protective role in bone destruction.

### Tomatidine Attenuated the Severity of Arthritis in CIA Rats

**FIGURE 3:**
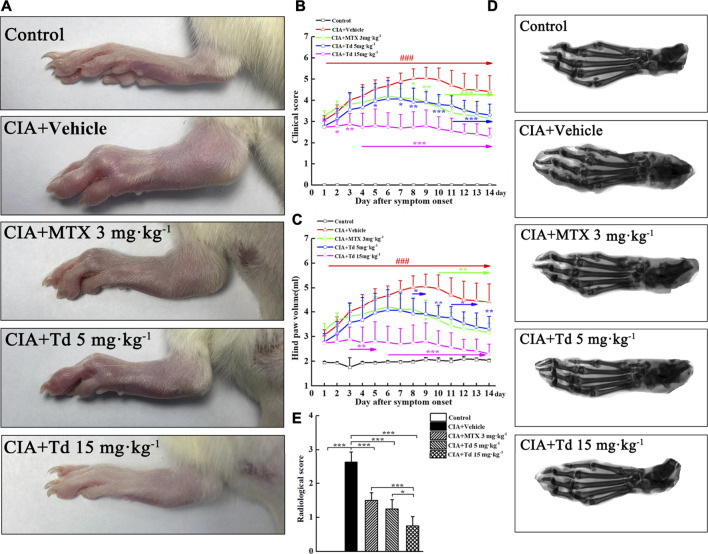
Td significantly suppressed synovial inflammation and bone destruction in CIA rats. **(A)** Representative photographs of the hind paws of CIA rats obtained from different groups. Clinical score **(B)** and hind paw volume **(C)** were inhibited by Td or MTX. *n* = 6, ^**###**^ *P* < 0.001 versus the normal control rats; ^*^ *P* < 0.05, ^**^ *P* < 0.01 and ^***^ *P* < 0.001 versus the vehicle-treated CIA rats. **(D)** Representative radiographs of the hind paws of CIA rats obtained from different groups. **(E)** Radiological scores were calculated as described in Materials and Methods section. *n* = 6, ^*^ *P* < 0.05 and ^***^ *P* < 0.001.

To assess the anti-arthritic effect of Td, a CIA model in Wistar rats was used. When the CIA model was firmly established (clinical score ≥2), Td with different doses or vehicle was intraperitoneally administrated once a day and continued for 14 days. As shown in Figure 3A, arthritis symptoms such as swelling and erythema were obviously observed in the vehicle-treated CIA rats, while Td or MTX treatment significantly attenuated the severity of the disease. Compared to MTX or 5 mg·kg^−1^ of Td which significantly suppressed the development of CIA, 15 mg·kg^−1^ of Td showed a stronger protective action as assessed by clinical score and paw swelling (Figures 3B,C).

### Tomatidine Suppressed Synovial Inflammation and Bone Destruction in CIA Rats

**FIGURE 4:**
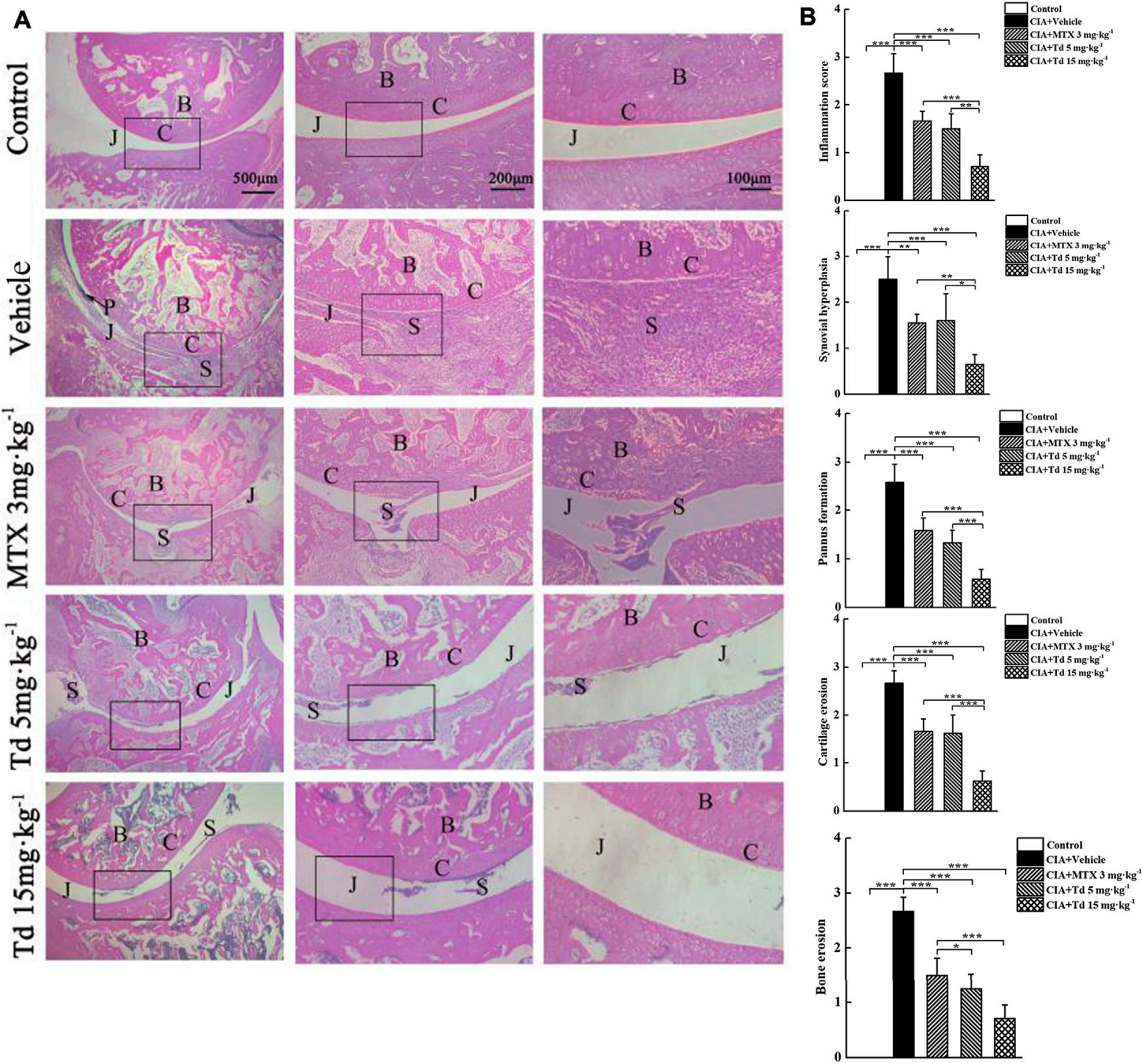
Effects of Td on histologic changes in CIA rats. **(A)** Representative H&E images of ankle joints obtained from different groups. **(B)** Histological scores were calculated as described in Materials and Methods section. *n* = 6, ^*^ *P* < 0.05, ^**^ *P* < 0.01 and ^***^ *P* < 0.001. B, bone; C, cartilage; J, joint space; P, pannus; S, synovium.

To further confirm the therapeutic effect of Td, radiographic and histopathological assessments were performed on the rat hind paws. The X-ray images showed that the typical RA changes, such as joint destruction and joint space narrowing, appeared in the vehicle-treated CIA rats without exception (Figure 3D). However, these destructions were significantly improved in MTX- or Td-treated groups. Furthermore, this improvement was more obvious in the high-dose Td group (Figure 3E). In line with the data of radiographic scoring and the clinical scoring, H&E staining and histopathological assessment showed that Td significantly attenuated the pathological characteristics of CIA rats including inflammatory cell infiltration, synovial hyperplasia, pannus formation, cartilage erosion, and bone erosion, which further demonstrated the inhibitory effect of Td on RA (Figures 4A,B).

To evaluate the potential adverse effect of Td on CIA rats, a preliminary study was carried out to analyze the weight loss, organ indexes and serum markers of liver injury (ALT and AST). As shown in Table 1, compared to the normal control, the vehicle-treated CIA rats showed a significant weight loss. However, this weight loss was significantly suppressed by Td treatment. Except that the kidney index was decreased by Td, the other organ indexes as well as serum ALT and AST have no significant differences between the Td-treated groups and the vehicle-treated CIA group, further supporting that the given doses of Td used in this study had no significant side effects.

**TABLE 1:** Effects of Td on organ indexes and serum ALT and AST in rats with collagen-induced arthritis.

Parameters	Control	Vehicle	MTX (3 mg·kg^−1^)	Td (5 mg·kg^−1^)	Td (15 mg·kg^−1^)
Body weight gain (%)	27.29 ± 5.98	−5.11 ± 1.35^**###**^	7.86 ± 2.47^***^	7.72 ± 2.44^***^	12.68 ± 3.66^***^
Index of heart (%)	0.34 ± 0.02	0.42 ± 0.03^**###**^	0.41 ± 0.05	0.37 ± 0.04	0.40 ± 0.01
Index of liver (%)	3.82 ± 0.25	4.76 ± 0.53^**##**^	3.88 ± 0.32^**^	4.38 ± 0.25	4.20 ± 0.55
Index of kidney (%)	0.39 ± 0.03	0.54 ± 0.06^**###**^	0.49 ± 0.04	0.44 ± 0.04^***^	0.45 ± 0.04^***^
Index of spleen (%)	0.25 ± 0.02	0.41 ± 0.06^**###**^	0.32 ± 0.05	0.37 ± 0.07	0.32 ± 0.06
Index of thymus (%)	0.26 ± 0.04	0.27 ± 0.06	0.23 ± 0.05	0.22 ± 0.06	0.29 ± 0.05
AST (U/L)	25.78 ± 5.81	23.90 ± 2.62	24.31 ± 2.00	24.62 ± 1.73	25.46 ± 0.92
ALT (U/L)	26.90 ± 0.48	27.72 ± 2.60	26.13 ± 0.48	25.38 ± 1.57	25.12 ± 1.65

Rats were intraperitoneally injected with Td (5 and 15 mg·kg^−1^) or 0.9% saline daily for up to 14 days. The MTX-treated group was intraperitoneally injected with MTX (3 mg·kg^−1^) every 3 days according to clinical usage. Data were expressed as mean *±* SD; *n* = 6, ^**##**^ *P* < 0.01 and ^**###**^ *P* < 0.001 versus the normal control (age-matched rats); ^**^ *P* < 0.01 and ^***^ *P* <0.001 versus the vehicle-treated CIA rats. ALT, alanine aminotransferase; AST, aspartate aminotransferase.

### Tomatidine Decreased Synthesis of Cytokines and MMP-9 in CIA Rats

**FIGURE 5:**
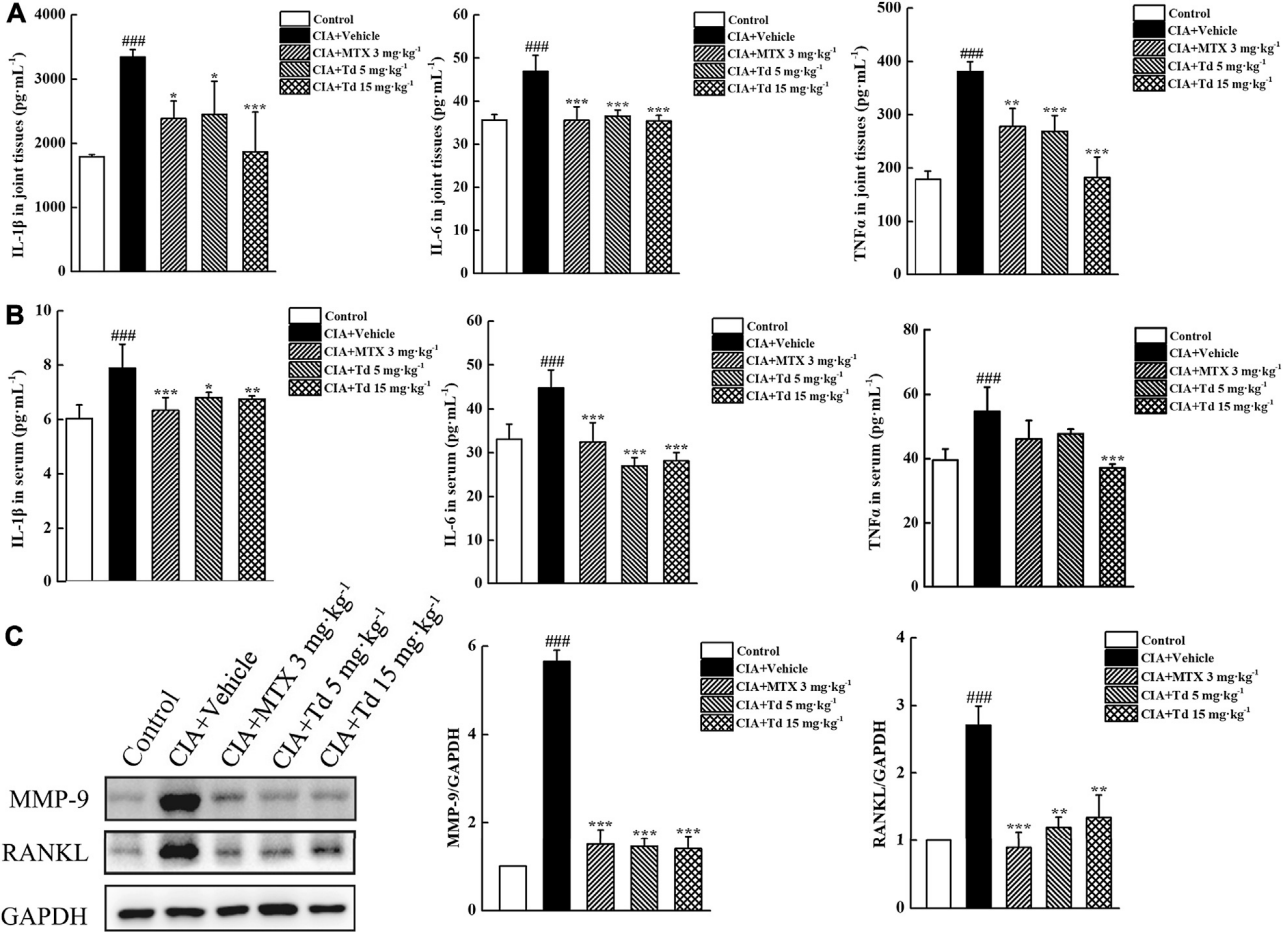
Td decreased synthesis of proinflammatory cytokines, MMP-9 and RANKL in CIA rats. **(A,B)** ELISA was performed to measure the protein levels of IL-1β, IL-6 and TNFα in serum and joint tissues of rats. *n* = 6, ^**###**^ *P* < 0.001 versus the normal control rats; ^*^ *P* < 0.01, ^**^ *P* < 0.05 and ^***^ *P* < 0.001 versus the vehicle-treated group. **(C)** Western blot was performed to examine the protein expression of MMP-9 and RANKL in joint tissues of rats. Relative expression was determined by densitometric analysis using ImageJ software. *n* = 6, ^**###**^ *P* < 0.001 versus the normal control rats; ^**^ *P* < 0.01 and ^***^ *P* < 0.001 versus the vehicle-treated group.

We have demonstrated that, in primary arthritic FLSs, Td could inhibit the expressions of multiple pro-inflammatory cytokines. To further confirm the *in vitro* results, serum and joint tissues of each group were collected and the protein expressions of IL-1β, IL-6 and TNFα were examined using ELISA. As shown in Figures 5A,B, the local and systemic levels of IL-1β, IL-6 and TNFα in the Td-treated groups were much lower than those in the vehicle-treated group. In addition, compared with the vehicle-treated rats, those treated with Td had significantly decreased production of MMP-9 and RANKL (Figure 5C), which further confirmed the data from arthritic FLSs.

### Tomatidine Inhibited TNFα-Induced MAPKs (ERK and JNK) and NF-κB Activations

**FIGURE 6:**
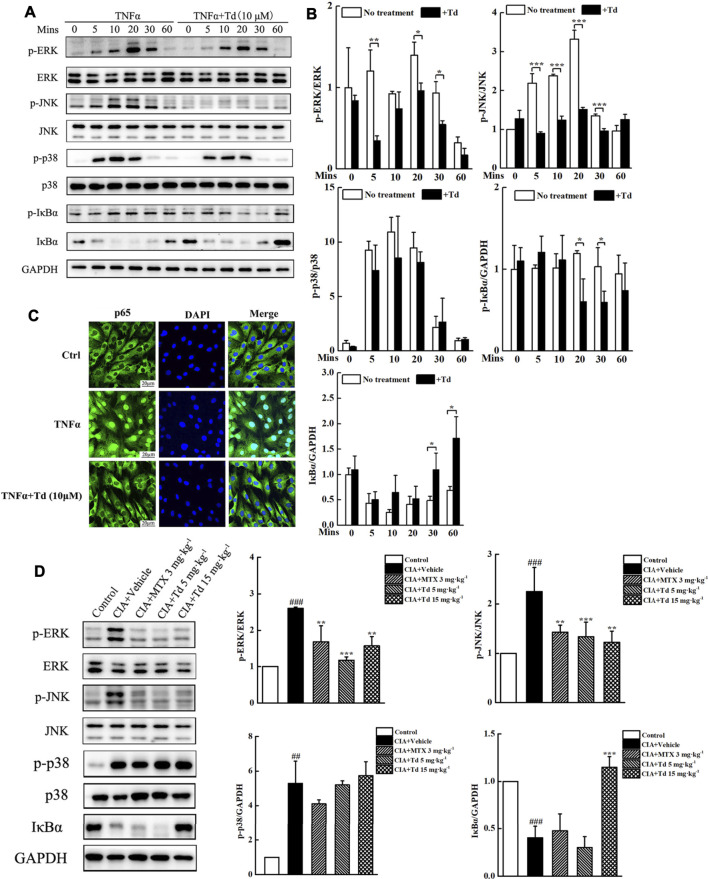
Td suppressed TNFα-induced MAPKs and NF-κB activations. **(A)** Arthritic FLSs were treated with or without Td (10 μM) for 4 h, and then incubated with TNFα (50 ng·mL^−1^) for 0, 5, 10, 20, 30 and 60 mins. Western blot was probed for p-ERK1/2, total ERK1/2, p-JNK1/2, total JNK1/2, p-p38, total p38, p-IκBα, IκBα and GAPDH. **(B)** The ratios of p-ERK/ERK, p-JNK/JNK, p-p38/p38, p-IκBα/GAPDH and IκBα/GAPDH were analyzed using ImageJ software. ^*^ *P* < 0.05, ^**^ *P* < 0.01 and ^***^ *P* < 0.001 versus TNFα-treated, Td-untreated cells. **(C)** Arthritic FLSs were seeded at a density of 2 × 10^4^/well in 24-well plates and cultured overnight. Following pre-treatment with or without Td (10 μM) for 4 h, and the cells were incubated with TNFα (50 ng·mL^−1^) for 30 min. The localization of p65 was fixed, stained, and visualized by immunofluorescence analysis. **(D)** The right hind paws were collected and joint tissue homogenates were used to measure the protein levels and phosphorylations of MAPK members (ERK, JNK and p38). Additionally, the protein level of IκBα was also detected. Relative expression was determined by densitometric analysis using ImageJ software. *n* = 6, ^**##**^ *P* < 0.01 and ^**###**^ *P* < 0.001 versus the normal control rats; ^**^ *P* < 0.01 and ^***^ *P* < 0.001 versus the vehicle-treated group.

To reveal the mechanisms through which Td plays an inhibitory action on CIA model and arthritic FLS cells, we used immunoblotting to examine the activations of MAPKs and NF-κB, which play vital roles in RA pathogenesis. In cultured primary arthritic FLSs, TNFα stimulation could rapidly increase the phosphorylation levels of the MAPK family members (ERK, JNK and p38) (Figures 6A,B). Td treatment significantly suppressed TNFα-induced phosphorylations of ERK and JNK but without effect on p38 (Figures 6A,B). In addition, TNFα stimulation initiated phosphorylation and degradation of IκBα. When the cells were treated with Td (10 μM), IκBα phosphorylation and degradation were both significantly suppressed (Figures 6A,B). It is well known that IκB degradation can liberate NF-κB p65 protein into the nucleus and trigger its downstream target gene expression (Makarov, 2001). Thus, we used immunofluorescence microscopy to detect the effect of Td on p65 nuclear translocation. As shown in Figure 6C, TNFα incubation for 30 min clearly promoted NF-κB p65 translocation from the cytoplasm to the nuclei. However, this translocation process was substantially blocked when the cell was treated with 10 μM Td, as evidenced by the cytoplasmic retention of p65 proteins (Figure 6C).

To confirm the *in vitro* results, we isolated the protein from the joint tissues of each group and examined the activations of MAPKs and NF-κB using Western blot. As expected, Td had no obvious influence on p38 phosphorylation. However Td significantly decreased the phosphorylation levels of ERK and JNK compared with the vehicle-treated group (Figure 6D). Additionally, the degradation of IκBα was also suppressed by high-dose Td treatment (Figure 6D).

## Discussion

Td, a steroidal alkaloid derived from the solanaceae family, has been reported to possess antioxidative, anti-inflammatory, anti-tumor, immunomodulatory and anti-osteoclastic properties (Lee et al., 2004; Chiu and Lin, 2008; Yan et al., 2013; Jeon and Kim, 2019). Based on the evidence, we reasoned that Td might exert a protective role in RA development. Indeed, this study demonstrated that Td could suppress synovial inflammatory and bone destruction through inhibiting the pathogenic behaviors of arthritic FLSs.

As the prominent cell type in synovium, FLSs play an indispensable role in establishing the complex three-dimensional synovial lining architecture. In inflamed RA synovium, FLS cells acquire and sustain uniquely aggressive properties including increasing proliferative and invasive capacity, escaping contacting inhibition and resisting apoptosis (Neidhart et al., 2003; Lahoti et al., 2014). These over-activated FLSs can autonomously drive and maintain joint inflammation through secretion of multiple pro-inflammatory mediators, directly invade and destroy articular cartilage through producing matrix metalloproteinases (MMPs), and promote bone erosion through synthesis of osteoclast differentiation factor RANKL (Takayanagi et al., 2000; Kim et al., 2009). Hence, inhibiting the pathogenic properties of FLSs may be a promising strategy for RA treatment. In this study, the *in vitro* and *in vivo* experiments provided rigorous demonstrations that Td could attenuate FLS’s destructive phenotypes. The EdU assay showed that Td significantly inhibited the proliferative potential of primary arthritic FLSs. In line with the *in vitro* data, the histopathological results showed that both low dose and high dose of Td could significantly suppress synovial hyperplasia in CIA rats. In addition, Td could dose-dependently reduce the migration rate of arthritic FLSs. The histopathological scores also demonstrated that the pannus formation in CIA rats was significantly suppressed by Td. MMPs, especially the members of MMP-2 and MMP-9, are well known to exert a critical role in ECM breakdown and tissues degradation (Gruber et al., 1996; Tchetverikov et al., 2004). MMP-2 is constitutively expressed in an inactive form in various cell types without being affected by pro-inflammatory mediators (Konttinen et al., 1999). However, MMP-9 is inducible by inflammatory cytokines, indicating a close relationship with synovial inflammation (Giannelli et al., 2004). In this study, Td had little effect on the production of MMP-2, however, it significantly suppressed synthesis of MMP-9. This finding was consistent with our *in vivo* results, which showed a protective effect of Td on cartilage erosion. In addition to MMP-9, Td also inhibited the expressions of IL-1β, IL-6 and TNFα at both the mRNA and protein levels, indicating that Td could suppress synovial inflammation. Indeed, the *in vivo* data showed that Td significantly attenuated inflammatory cell infiltration and inflammation response. Notably, RANKL, an essential inducer of osteoclastogenesis, was significantly down-regulated by Td. Consistent with this result, the radiographic evaluation and the histopathological analysis both verified that Td at the dosages of 5 mg·kg^−1^ and 15 mg·kg^−1^ effectively protected against bone destruction in CIA rats. The previous study reported that Td could directly inhibited osteoclast formation through modulating multiple TRAF6-mediated pathways (Hu et al., 2019). Therefore, we speculated that Td’s anti-bone erosive action might attribute to its direct inhibition on osteoclastogenesis, and an indirect inhibitory effect through decreasing RANKL expression in arthritic FLSs.

**FIGURE 7:**
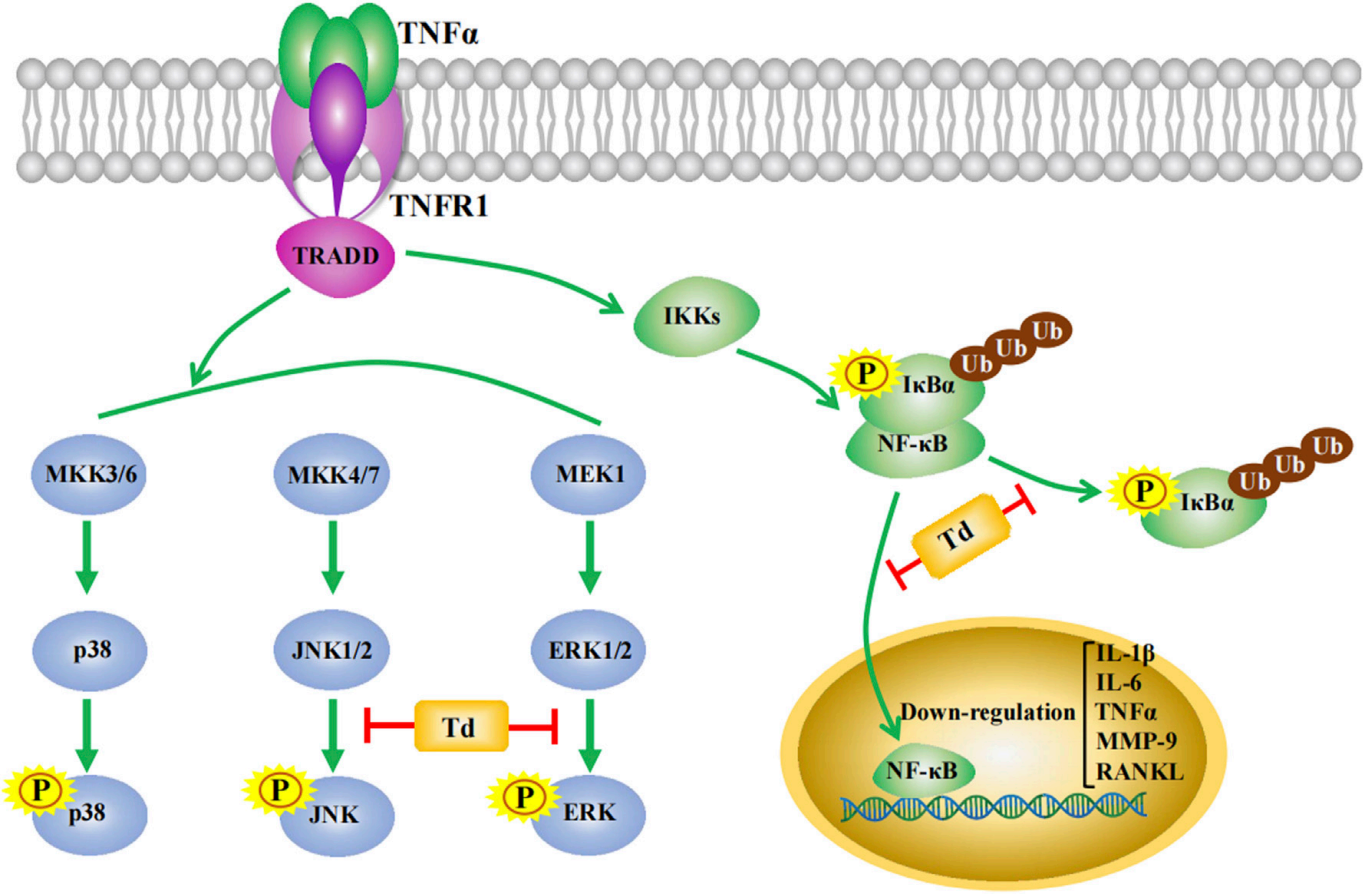
Schematic summarizing of the mechanism by which Td relieved the destructive behaviors of FLSs and prevented the development of CIA. In TNFα-stimulated FLSs and CIA rat model, MAPK and NF-κB signaling pathways were activated. Td treatment significantly inhibited phosphorylations of ERK and JNK. Also, NF-κB activation was suppressed *via* decreasing IκBα phosphorylation and degradation, and thereby inhibiting the nuclear translocation of the p65 subunit of NF-κB. Accordingly, NF-κB target genes including *IL-1β*, *IL-6*, *TNFα, MMP-9* and *RANKL* were downregulated.

Having demonstrated that Td exerted an inhibitory action on CIA rats and arthritic FLSs, we further analyzed the possibly involved molecular mechanisms. MAPKs are well known to play essential roles in regulating multiple cellular events such as cell migration, proliferation and secretion of proinflammatory cytokines (Herlaar and Brown, 1999; Han and Sun, 2007; Bogoyevitch et al., 2010). In the over-activated RA FLSs, the three MAPK kinases (ERK, JNK and p38) showed significantly up-regulated phosphorylation and activation. Therefore, the blockage of their activations may offer promising benefits to RA treatment (Thompson and Lyons, 2005; Senolt et al., 2009). In arthritic FLSs, we found that Td had little effect on the phosphorylation of p38 but significantly attenuated TNFa-induced phosphorylation of ERK and JNK. A similar result was observed in the CIA rat model, which further confirmed the inhibition of Td on ERK and JNK pathways. Besides, Td also suppressed the activation of NF-κB, as evidenced by the downregulations of IκBα phosphorylation and degradation, and thereby inhibition of p65 nuclear translocation. These results were further demonstrated by the real-time PCR data that Td significantly inhibited the transcripts of NF-κB target genes including *IL-1β*, *IL-6*, *TNFα*, *MMP-9* and *RANKL*. Taken together, these data indicated that Td might play an inhibitory action on RA via multiple targets (Figure 7), and further studies are needed to clarify its direct binding sites.

In summary, we here demonstrated for the first time that Td could inhibit the pathological properties of arthritic FLSs *in vitro* and protect against CIA *in vivo*. This inhibitory effect of Td on RA might attribute to the decreased activations of MAPKs (ERK and JNK) and NF-κB. Td has the potential to become a complementary or alternative medicine for RA therapy.

## Data Availability

The raw data supporting the conclusion of this article will be made available by the authors, without undue reservation, to any qualified researcher.

## Abbreviations

Td, tomatidine; RA, rheumatoid arthritis; TNFα, tumor necrosis factor alpha; FLSs, fibroblast-like synoviocytes; CIA, collagen-induced arthritis; IL-1β, Interleukin-1β; IL-6, Interleukin-6; RANKL, receptor activator of nuclear factor κB ligand; MMP-9, matrix metallopeptidase nine; MAPK, mitogen-activated protein kinase; ERK, extracellular signal-regulated kinase; JNK, c-Jun N-terminal kinases; NF-κB, nuclear factor of kappaB.

## References

[B1] Ath A. J. F. Denton J. (1991). Atlas of Synovial Fluid Cytopathology. Springer Netherlands.

[B2] Athanasou N. A. (1996). Cellular Biology of Bone-Resorbing Cells. J. Bone Jt. Surg Am 78 (7), 1096–1112. Epub 1996/07/01PubMed PMID: 8698729. 10.2106/00004623-199607000-00016 8698729

[B3] Bartok B. Firestein G. S. (2010). Fibroblast-like Synoviocytes: Key Effector Cells in Rheumatoid Arthritis. Immunol. Rev. 233 (1), 233–255. Epub 2010/03/03PubMed PMID: 20193003; PubMed Central PMCID: PMCPMC2913689. 10.1111/j.0105-2896.2009.00859.x 20193003 PMC2913689

[B4] Bogoyevitch M. A. Ngoei K. R. Zhao T. T. Yeap Y. Y. Ng D. C. (2010). c-Jun N-Terminal Kinase (JNK) Signaling: Recent Advances and Challenges. Biochim. Biophys. Acta 1804 (3), 463–475. Epub 2009/11/11PubMed PMID: 19900593. 10.1016/j.bbapap.2009.11.002 19900593

[B5] Cai X. Zhou H. Wong Y. F. Xie Y. Liu Z. Q. Jiang Z. H. (2007). Suppression of the Onset and Progression of Collagen-Induced Arthritis in Rats by QFGJS, a Preparation from an Anti-arthritic Chinese Herbal Formula. J. Ethnopharmacol 110 (1), 39–48. Epub 2006/10/20PubMed PMID: 17049776. 10.1016/j.jep.2006.09.008 17049776

[B6] Chiu F. L. Lin J. K. (2008). Tomatidine Inhibits iNOS and COX-2 through Suppression of NF-kappaB and JNK Pathways in LPS-Stimulated Mouse Macrophages. FEBS Lett. 582 (16), 2407–2412. Epub 2008/06/12PubMed PMID: 18544347. 10.1016/j.febslet.2008.05.049 18544347

[B7] Danks L. Sabokbar A. Gundle R. Athanasou N. A. (2002). Synovial Macrophage-Osteoclast Differentiation in Inflammatory Arthritis. Ann. Rheum. Dis. 61 (10), 916–921. Epub 2002/09/14PubMed PMID: 12228163; PubMed Central PMCID: PMCPMC1753924. 10.1136/ard.61.10.916 12228163 PMC1753924

[B8] Dyle M. C. Ebert S. M. Cook D. P. Kunkel S. D. Fox D. K. Bongers K. S. (2014). Systems-based Discovery of Tomatidine as a Natural Small Molecule Inhibitor of Skeletal Muscle Atrophy. J. Biol. Chem. 289 (21), 14913–14924. Epub 2014/04/11PubMed PMID: 24719321; PubMed Central PMCID: PMCPMC4031541. 10.1074/jbc.M114.556241 24719321 PMC4031541

[B9] Firestein G. S. (2003). Evolving Concepts of Rheumatoid Arthritis. Nature 423 (6937), 356–361. Epub 2003/05/16PubMed PMID: 12748655. 10.1038/nature01661 12748655

[B10] Friedman M. Levin C. E. Lee S. U. Kim H. J. Lee I. S. Byun J. O. (2009). Tomatine-containing green Tomato Extracts Inhibit Growth of Human Breast, colon, Liver, and Stomach Cancer Cells. J. Agric. Food Chem. 57 (13), 5727–5733. Epub 2009/06/12PubMed PMID: 19514731. 10.1021/jf900364j 19514731

[B11] Fujiwara Y. Kiyota N. Tsurushima K. Yoshitomi M. Horlad H. Ikeda T. (2012). Tomatidine, a Tomato Sapogenol, Ameliorates Hyperlipidemia and Atherosclerosis in apoE-Deficient Mice by Inhibiting Acyl-CoA:cholesterol Acyl-Transferase (ACAT). J. Agric. Food Chem. 60 (10), 2472–2479. Epub 2012/01/10PubMed PMID: 22224814. 10.1021/jf204197r 22224814

[B12] Giannelli G. Erriquez R. Iannone F. Marinosci F. Lapadula G. Antonaci S. (2004). MMP-2, MMP-9, TIMP-1 and TIMP-2 Levels in Patients with Rheumatoid Arthritis and Psoriatic Arthritis. Clin. Exp. Rheumatol. 22 (3), 335–338. Epub 2004/05/18. PubMed PMID: 15144129. 15144129

[B13] Gravallese E. M. (2002). Bone Destruction in Arthritis. Ann. Rheum. Dis. 61 Suppl 2 (Suppl. 2Suppl 2), ii84–6. Epub 2002/10/16PubMed PMID: 12379632; PubMed Central PMCID: PMCPMC1766721. 10.1136/ard.61.suppl_2.ii84 12379632 PMC1766721

[B14] Gruber B. L. Sorbi D. French D. L. Marchese M. J. Nuovo G. J. Kew R. R. (1996). Markedly Elevated Serum MMP-9 (Gelatinase B) Levels in Rheumatoid Arthritis: a Potentially Useful Laboratory Marker. Clin. Immunol. Immunopathol 78 (2), 161–171. Epub 1996/02/01PubMed PMID: 8625558. 10.1006/clin.1996.0025 8625558

[B15] Han J. Sun P. (2007). The Pathways to Tumor Suppression via Route P38. Trends Biochem. Sci. 32 (8), 364–371. Epub 2007/07/13PubMed PMID: 17624785. 10.1016/j.tibs.2007.06.007 17624785

[B16] Herlaar E. Brown Z. (1999). p38 MAPK Signalling Cascades in Inflammatory Disease. Mol. Med. Today 5 (10), 439–447. Epub 1999/09/28PubMed PMID: 10498912. 10.1016/s1357-4310(99)01544-0 10498912

[B17] Hu B. Sun X. Yang Y. Ying Z. Meng J. Zhou C. (2019). Tomatidine Suppresses Osteoclastogenesis and Mitigates Estrogen Deficiency-Induced Bone Mass Loss by Modulating TRAF6-Mediated Signaling. FASEB J. 33 (2), 2574–2586. Epub 2018/10/05PubMed PMID: 30285579. 10.1096/fj.201800920R 30285579

[B18] Jeon S. Kim M. M. (2019). Tomatidine Inhibits Cell Invasion through the Negative Modulation of Gelatinase and Inactivation of P38 and ERK. Chem. Biol. Interact 313, 108826. Epub 2019/09/24PubMed PMID: 31545954. 10.1016/j.cbi.2019.108826 31545954

[B19] Kim K. W. Cho M. L. Oh H. J. Kim H. R. Kang C. M. Heo Y. M. (2009). TLR-3 Enhances Osteoclastogenesis through Upregulation of RANKL Expression from Fibroblast-like Synoviocytes in Patients with Rheumatoid Arthritis. Immunol. Lett. 124 (1), 9–17. Epub 2009/05/19PubMed PMID: 19446344. 10.1016/j.imlet.2009.02.006 19446344

[B20] Kobayashi K. Takahashi N. Jimi E. Udagawa N. Takami M. Kotake S. (2000). Tumor Necrosis Factor Alpha Stimulates Osteoclast Differentiation by a Mechanism Independent of the ODF/RANKL-RANK Interaction. J. Exp. Med. 191 (2), 275–286. Epub 2000/01/19PubMed PMID: 10637272; PubMed Central PMCID: PMCPMC2195746. 10.1084/jem.191.2.275 10637272 PMC2195746

[B21] Konttinen Y. T. Ainola M. Valleala H. Ma J. Ida H. Mandelin J. (1999). Analysis of 16 Different Matrix Metalloproteinases (MMP-1 to MMP-20) in the Synovial Membrane: Different Profiles in Trauma and Rheumatoid Arthritis. Ann. Rheum. Dis. 58 (11), 691–697. Epub 1999/10/26PubMed PMID: 10531073; PubMed Central PMCID: PMCPMC1752794. 10.1136/ard.58.11.691 10531073 PMC1752794

[B22] Lahoti T. S. Hughes J. M. Kusnadi A. John K. Zhu B. Murray I. A. (2014). Aryl Hydrocarbon Receptor Antagonism Attenuates Growth Factor Expression, Proliferation, and Migration in Fibroblast-like Synoviocytes from Patients with Rheumatoid Arthritis. J. Pharmacol. Exp. Ther. 348 (2), 236–245. Epub 2013/12/07PubMed PMID: 24309559; PubMed Central PMCID: PMCPMC3912548. 10.1124/jpet.113.209726 24309559 PMC3912548

[B23] Lange F. Bajtner E. Rintisch C. Nandakumar K. S. Sack U. Holmdahl R. (2005). Methotrexate Ameliorates T Cell Dependent Autoimmune Arthritis and Encephalomyelitis but Not Antibody Induced or Fibroblast Induced Arthritis. Ann. Rheum. Dis. 64 (4), 599–605. Epub 2004/09/04PubMed PMID: 15345503; PubMed Central PMCID: PMCPMC1755430. 10.1136/ard.2004.026120 15345503 PMC1755430

[B24] Laragione T. Brenner M. Mello A. Symons M. Gulko P. S. (2008). The Arthritis Severity Locus Cia5d Is a Novel Genetic Regulator of the Invasive Properties of Synovial Fibroblasts. Arthritis Rheum. 58 (8), 2296–2306. Epub 2008/08/01PubMed PMID: 18668563; PubMed Central PMCID: PMCPMC2714698. 10.1002/art.23610 18668563 PMC2714698

[B25] Lee K. R. Kozukue N. Han J. S. Park J. H. Chang E. Y. Baek E. J. (2004). Glycoalkaloids and Metabolites Inhibit the Growth of Human colon (HT29) and Liver (HepG2) Cancer Cells. J. Agric. Food Chem. 52 (10), 2832–2839. Epub 2004/05/13PubMed PMID: 15137822. 10.1021/jf030526d 15137822

[B26] Lee S. T. Wong P. F. Cheah S. C. Mustafa M. R. (2011). Alpha-tomatine Induces Apoptosis and Inhibits Nuclear Factor-Kappa B Activation on Human Prostatic Adenocarcinoma PC-3 Cells. PloS one 6 (4), e18915. Epub 2011/05/05PubMed PMID: 21541327; PubMed Central PMCID: PMCPMC3082542. 10.1371/journal.pone.0018915 21541327 PMC3082542

[B27] Liu C. Zhang Y. Kong X. Zhu L. Pang J. Xu Y. (2013). Triptolide Prevents Bone Destruction in the Collagen-Induced Arthritis Model of Rheumatoid Arthritis by Targeting RANKL/RANK/OPG Signal Pathway. Evidence-Based Complement. Altern. Med., 2013, 1–12. Epub 2013/04/11PubMed PMID: 23573139; PubMed Central PMCID: PMCPMC3610373. 10.1155/2013/626038 PMC3610373 23573139

[B28] Liu M. Zhou X. Zhou L. Liu Z. Yuan J. Cheng J. (2018). Carnosic Acid Inhibits Inflammation Response and Joint Destruction on Osteoclasts, Fibroblast-like Synoviocytes, and Collagen-Induced Arthritis Rats. J. Cel Physiol 233 (8), 6291–6303. Epub 2018/03/10PubMed PMID: 29521424. 10.1002/jcp.26517 29521424

[B29] Liu Z. Zhou L. Ma X. Sun S. Qiu H. Li H. (2018). Inhibitory Effects of Tubeimoside I on Synoviocytes and Collagen-Induced Arthritis in Rats. J. Cel Physiol 233 (11), 8740–8753. Epub 2018/05/16PubMed PMID: 29761884. 10.1002/jcp.26754 29761884

[B30] Ma Y. Pope R. M. (2005). The Role of Macrophages in Rheumatoid Arthritis. Curr. Pharm. Des. 11 (5), 569–580. Epub 2005/02/22PubMed PMID: 15720276. 10.2174/1381612053381927 15720276

[B31] Makarov S. S. (2001). NF-kappa B in Rheumatoid Arthritis: a Pivotal Regulator of Inflammation, Hyperplasia, and Tissue Destruction. Arthritis Res. 3 (4), 200–206. Epub 2001/07/05PubMed PMID: 11438035; PubMed Central PMCID: PMCPMC128895. 10.1186/ar300 11438035 PMC128895

[B32] Mor A. Abramson S. B. Pillinger M. H. (2005). The Fibroblast-like Synovial Cell in Rheumatoid Arthritis: a Key Player in Inflammation and Joint Destruction. Clin. Immunol. 115 (2), 118–128. Epub 2005/05/12PubMed PMID: 15885632. 10.1016/j.clim.2004.12.009 15885632

[B33] Neidhart M. Seemayer C. A. Hummel K. M. Michel B. A. Gay R. E. Gay S. (2003). Functional Characterization of Adherent Synovial Fluid Cells in Rheumatoid Arthritis: Destructive Potential *In Vitro* and *In Vivo* . Arthritis Rheum. 48 (7), 1873–1880. Epub 2003/07/09PubMed PMID: 12847681. 10.1002/art.11166 12847681

[B34] Noss E. H. Brenner M. B. (2008). The Role and Therapeutic Implications of Fibroblast-like Synoviocytes in Inflammation and Cartilage Erosion in Rheumatoid Arthritis. Immunol. Rev. 223, 252–270. Epub 2008/07/11PubMed PMID: 18613841. 10.1111/j.1600-065X.2008.00648.x 18613841

[B35] Qiu H. Sun S. Ma X. Cui C. Chen G. Liu Z. (2018). Jatrorrhizine Hydrochloride Suppresses Proliferation, Migration, and Secretion of Synoviocytes *In Vitro* and Ameliorates Rat Models of Rheumatoid Arthritis *In Vivo* . Int. J. Mol. Sci. 19 (5). PubMed PMID: 29783696; PubMed Central PMCID: PMCPMC5983572. 10.3390/ijms19051514 Epub 2018/05/23 PMC5983572 29783696

[B36] Senolt L. Vencovský J. Pavelka K. Ospelt C. Gay S. (2009). Prospective New Biological Therapies for Rheumatoid Arthritis. Autoimmun. Rev. 9 (2), 102–107. Epub 2009/03/31PubMed PMID: 19328245. 10.1016/j.autrev.2009.03.010 19328245

[B37] Shieh J. M. Cheng T. H. Shi M. D. Wu P. F. Chen Y. Ko S. C. (2011). α-Tomatine Suppresses Invasion and Migration of Human Non-small Cell Lung Cancer NCI-H460 Cells through Inactivating FAK/PI3K/Akt Signaling Pathway and Reducing Binding Activity of NF-Κb. Cell Biochem. Biophys. 60 (3), 297–310. Epub 2011/01/26PubMed PMID: 21264526. 10.1007/s12013-011-9152-1 21264526

[B38] Skapenko A. Leipe J. Lipsky P. E. Schulze-Koops H. (2005). The Role of the T Cell in Autoimmune Inflammation. Arthritis Res. Ther. 7 Suppl 2 (Suppl. 2Suppl 2), S4–S14. Epub 2005/04/19PubMed PMID: 15833146; PubMed Central PMCID: PMCPMC2833981. 10.1186/ar1703 15833146 PMC2833981

[B39] Song S. E. Shin S. K. Cho H. W. Im S. S. Bae J. H. Woo S. M. (2018). Tomatidine Inhibits Tumor Necrosis Factor-α-Induced Apoptosis in C2C12 Myoblasts via Ameliorating Endoplasmic Reticulum Stress. Mol. Cel. Biochem. 444 (1-2), 17–25. Epub 2017/12/03PubMed PMID: 29196971. 10.1007/s11010-017-3226-3 29196971

[B40] Takayanagi H. Iizuka H. Juji T. Nakagawa T. Yamamoto A. Miyazaki T. (2000). Involvement of Receptor Activator of Nuclear Factor kappaB Ligand/osteoclast Differentiation Factor in Osteoclastogenesis from Synoviocytes in Rheumatoid Arthritis. Arthritis Rheum. 43 (2), 259–269. Epub 2000/02/29PubMed PMID: 10693864. 10.1002/1529-013110.1002/1529-0131(200002)43:2<259::AID-ANR4>3.0.CO;2-W 10693864

[B41] Tchetverikov I. Ronday H. K. Van El B. Kiers G. H. Verzijl N. TeKoppele J. M. (2004). MMP Profile in Paired Serum and Synovial Fluid Samples of Patients with Rheumatoid Arthritis. Ann. Rheum. Dis. 63 (7), 881–883. Epub 2004/06/15PubMed PMID: 15194590; PubMed Central PMCID: PMCPMC1755080. 10.1136/ard.2003.013243 15194590 PMC1755080

[B42] Thompson N. Lyons J. (2005). Recent Progress in Targeting the Raf/MEK/ERK Pathway with Inhibitors in Cancer Drug Discovery. Curr. Opin. Pharmacol. 5 (4), 350–356. Epub 2005/06/16PubMed PMID: 15955734. 10.1016/j.coph.2005.04.007 15955734

[B43] Ueland T. Yndestad A. Øie E. Florholmen G. Halvorsen B. Frøland S. S. (2005). Dysregulated Osteoprotegerin/RANK Ligand/RANK axis in Clinical and Experimental Heart Failure. Circulation 111 (19), 2461–2468. Epub 2005/05/11PubMed PMID: 15883214. 10.1161/01.Cir.0000165119.62099.14 15883214

[B44] Yan K. H. Lee L. M. Yan S. H. Huang H. C. Li C. C. Lin H. T. (2013). Tomatidine Inhibits Invasion of Human Lung Adenocarcinoma Cell A549 by Reducing Matrix Metalloproteinases Expression. Chem. Biol. Interact 203 (3), 580–587. Epub 2013/04/10PubMed PMID: 23566884. 10.1016/j.cbi.2013.03.016 23566884

[B45] Yasuda H. Shima N. Nakagawa N. Yamaguchi K. Kinosaki M. Mochizuki S. (1998). Osteoclast Differentiation Factor Is a Ligand for Osteoprotegerin/osteoclastogenesis-Inhibitory Factor and Is Identical to TRANCE/RANKL. Proc. Natl. Acad. Sci. U S A. 95 (7), 3597–3602. Epub 1998/05/09PubMed PMID: 9520411; PubMed Central PMCID: PMCPMC19881. 10.1073/pnas.95.7.3597 9520411 PMC19881

[B46] Yuan H. Y. Zhang X. L. Zhang X. H. Meng L. Wei J. F. (2015). Analysis of Patents on Anti-rheumatoid Arthritis Therapies Issued in China. Expert Opin. Ther. Pat 25 (8), 909–930. Epub 2015/06/13PubMed PMID: 26066366. 10.1517/13543776.2015.1044972 26066366

